# Endovascular Treatment of a Bilateral Phlegmasia Caerulea Dolens in a Two Year Old Child With Inferior Vena Cava Atresia: Case Report and Literature Review

**DOI:** 10.1016/j.ejvsvf.2025.07.002

**Published:** 2025-07-08

**Authors:** Marco Fresa, Aurel Vassili Njami, Ophélie De Pury, Rafael Trunfio, Mattia Rizzi, Adrien De Cock, Guillaume Maître, Lucia Mazzolai

**Affiliations:** a Angiology Department, Centre for Malformation and Rare Vascular Diseases, Lausanne University Hospital (CHUV), Lausanne, Switzerland; b Paediatric Intensive Care Department, Lausanne University Hospital (CHUV), Lausanne, Switzerland; c Vascular Surgery Department, Lausanne University Hospital (CHUV), Lausanne, Switzerland; d Haematology Department, Lausanne University Hospital (CHUV), Lausanne, Switzerland; e Anaesthetics Department, Lausanne University Hospital (CHUV), Lausanne, Switzerland

**Keywords:** Acute deep vein thrombosis, Endovascular, Phlegmasia, Vena cava atresia, Venous

## Abstract

**Objective:**

Phlegmasia caerulea dolens (PCD) is a rare and potentially life threatening complication of deep vein thrombosis. It is more commonly reported in adult patients and rarely in the paediatric population, especially in healthy children. This review describes the case of a child with PCD and reviews the few cases of paediatric PCD previously reported in the literature.

**Methods:**

A search of the Medline database was performed with the purpose of identifying other similar cases and treatment modalities.

**Results:**

This study describes the case of a child with PCD in the setting of an atretic inferior vena cava, who was successfully treated with endovascular mechanical thrombectomy (Indigo Lightning Penumbra aspiration catheter) and intraluminal recanalisation and venoplasty of the inferior vena cava, with full recovery and no sequelae after 12 months of follow up.

**Conclusion:**

The use of mechanical thrombectomy in the setting of severe acute deep vein thrombosis is well established in adults, but not in children. Moreover, the recanalisation of an atretic inferior vena cava in such a young patient has not been described previously. The clinical outcome of this threatening condition was favourable, confirming that modern endovascular techniques for the venous system may also apply to the paediatric population and help deal with similar situations in this subset of patients.

## HIGHLIGHTS

• Phlegmasia caerulea dolens is a rare DVT complication causing acute limb ischaemia.
• Inferior vena cava (IVC) atresia is a rare condition that is often discovered in young adults.
• A child with PCD and atretic IVC was successfully treated by thrombectomy and IVC recanalisation.
• Endovascular treatment of paediatric PCD with IVC atresia can provide good outcomes.

## INTRODUCTION

Phlegmasia caerulea dolens (PCD) is a rare but serious complication of deep vein thrombosis (DVT) that in its most severe form can lead to acute limb ischaemia due to increased post-capillary pressure and flow obstruction.^1^ Clinical presentation varies from mottled skin with marked oedema, pain, coldness, cyanosis, and impaired capillary refill time to purpuric skin discoloration and gangrene. PCD is more commonly reported in adults and rarely in the paediatric population. PCD is an exceptionally rare condition in healthy children, as reflected by the low incidence rate of venous thromboembolism in the paediatric population.^2^

This review reports the case of a two year old girl with haemolytic anaemia and a previously unknown atretic inferior vena cava (IVC) who developed severe bilateral PCD secondary to a bilateral iliofemoral DVT, and who was successfully treated with endovascular mechanical thrombectomy and recanalisation of the IVC.

## CASE REPORT

A two year old girl with no medical history presented with marked pallor, jaundice, a decrease in food intake, and increased fatigue over 48 hours.

She was born at term, with no intensive care unit stay at birth or umbilical catheter placement. On admission, the work up revealed severe regenerative haemolytic anaemia and the Coombs test was positive with warm antibodies. Corticosteroid treatment was started immediately. On day 7, she complained of upper back and bilateral leg pain. Her legs appeared slightly swollen with bilateral discolouration. Acute haemolytic crisis and lower limb thrombosis were suspected. Her condition deteriorated rapidly over a couple of hours, as she became apathetic, diaphoretic, with a tachycardia up to 180 bpm, tachypnoea, and hypothermia at 35.5 °C. Her lower limbs became erythematous to bluish with severe swelling and induration.

**Figure 1:**
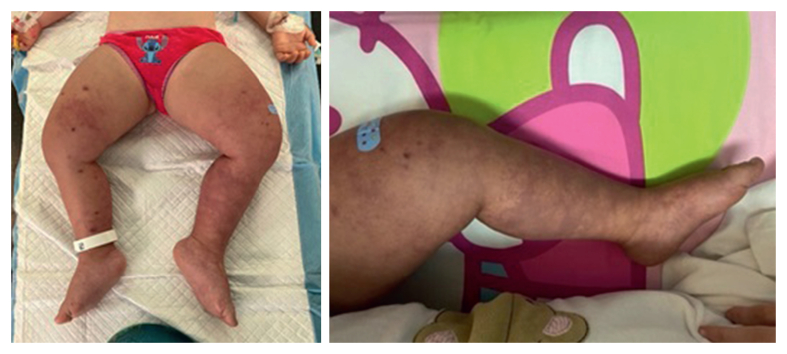
Bilateral phlegmasia caerula dolens. A - Massive oedema and ischaemic aspect of the lower limbs at the time of surgery. B - Clinical aspect of left lower limb at diagnosis.

In the intensive care unit (ICU), she presented with tachycardia, mottled and cold extremities, weak peripheral pulses, and acute renal failure with anuria. The appearance of her lower extremities changed to PCD (Fig. 1).

**Figure 2:**
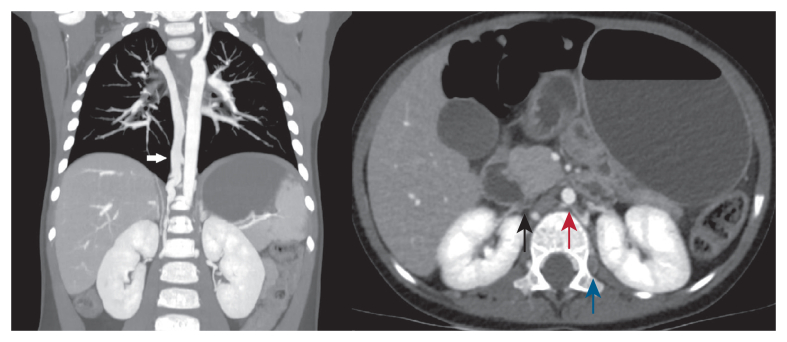
Computed tomography scan showing (A) the atretic inferior vena cava (black arrow), aorta (red arrow), and thrombosed lumbar vein (blue arrow) and (B) the dilated azygos system (white arrow).

A duplex ultrasound study demonstrated absence of the infrarenal vena cava and complete thrombotic occlusion of both iliofemoral and popliteal veins, confirming a diagnosis of bilateral lower extremity PCD. The arterial flow in the lower limbs was highly resistive, and the toe pressure was reduced with a bilateral toe brachial index of 0.30. At the same time, a cardiac ultrasound showed non-dilated right heart chambers with normal right ventricular systolic function. A computed tomography scan showed complete bilateral iliofemoral venous thrombosis up to the common iliac confluence and the lumbar veins from T12 and L2. The IVC appeared absent (Fig. 2B), with compensatory dilatation of the lumbar, hemi-azygos, and azygos systems into which both renal veins drained. The azygos vein in the thorax was the same size as the thoracic aorta, confirming a chronic compensatory anomaly (Fig. 2A). There was no evidence of pulmonary embolism or intracranial haemorrhage.

Due to the rapid onset and progression of phlegmasia and haemodynamic shock, a multidisciplinary team decided to urgently perform endovascular thrombectomy.

The patient had lactic acidosis (pH 7.1, lactate 2.5 mmol/L), was anaemic, had renal insufficiency with high potassium (5.7 mmol/L) and high CK levels (25 000 U/L) indicating rhabdomyolysis. The procedure was performed under general anaesthesia in the prone position and vasoactive support with norepinephrine was necessary throughout the procedure.

**Figure 3:**
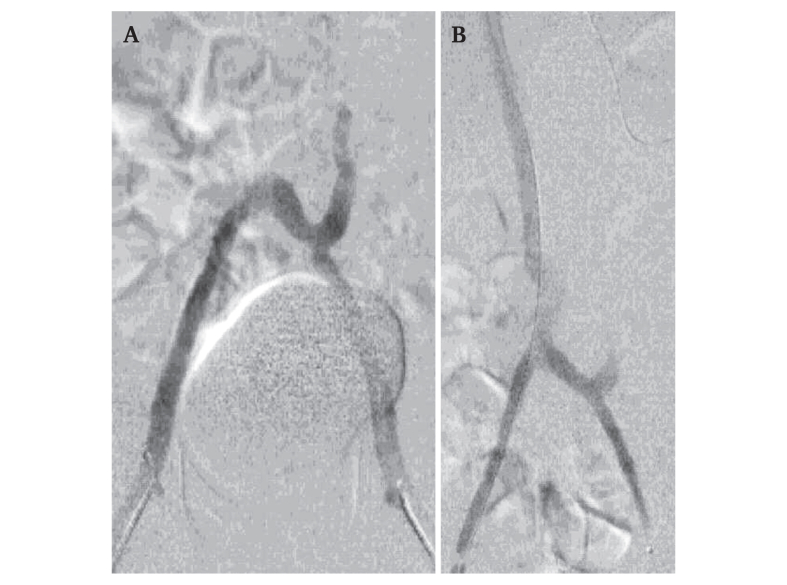
Intra-operative phlebographic images showing (A) bilateral iliac patency after thrombectomy, with no outflow even after main lumbar vein thrombectomy and (B) result after inferior vena cava angioplasty.

Bilateral 4 F sheath popliteal vein access was obtained. The initial venogram confirmed complete and extensive thrombosis of the femoral veins with no contrast passing cephalad to the occlusions. After upgrading the sheaths, a 7 F Indigo Lightning Penumbra aspiration catheter (Penumbra Inc., Alameda, CA, USA) was inserted. After one aspiration cycle on each side, all thrombotic material was aspirated with a blood loss of approximately 180 mL. Venography showed patent veins but no outflow due to thrombosis of the lumbar veins and the absence of the IVC, even after aspiration of the left main ascending lumbar vein. Intravascular recanalisation of the absent IVC was technically challenging and took more than two hours. A lateral view confirmed the anterior positioning of the guidewire in relation to the vertebrae, in a straight line. Progressive angioplasty of the vena cava was performed with 3–10 mm diameter balloons, requiring up to 30 atmospheres of inflation pressure, restoring a patent IVC. The completion venogram showed a regular IVC in the retro hepatic and inter renal segment (Fig. 3). The patient's heart rate responded rapidly and favourably after opening the IVC, confirming the impaired preload as a concurrent cause of shock.

Catheter directed thrombolysis was initiated through the popliteal sheaths with recombinant tissue plasminogen activator (Alteplase; Genentech, San Francisco, CA, USA) at 0.1 mg/h divided between the two catheters for overnight infusion. Fibrinogen levels were checked every four hours while the patient was monitored in the paediatric ICU. After the patient was repositioned supine, venous duplex showed good spontaneous flow in the iliofemoral veins.

No organ dysfunction was seen post-operatively. Perfusion of the lower limbs improved dramatically overnight, with a reduction in oedema and gradual recovery of normal skin colour and palpable pulses. Kidney failure immediately improved after the procedure.

Twelve hours later, the duplex scan showed patency of all veins from popliteal to the right atrium. The toe pressure and arterial wave pattern normalised bilaterally. The low dose catheter directed thrombolysis (CDT) was stopped after 12 hours. A favourable evolution marked the rest of the clinical course. She was extubated the day following the procedure. Corticosteroids were continued with prednisolone 2 mg/kg/day for the haemolytic anaemia. She required one platelet transfusion and one erythrocyte transfusion. The lower limbs improved day by day.

The thrombophilia work up was negative. There was only a positive Coombs test with non-specific warm antibodies. Covid-19, parvovirus B19, cytomegalovirus and Epstein–Barr virus serologies were negative. The autoimmune work up for lymphocyte count and immunoglobulin assays was normal. There were no anti-nuclear antibodies for lupus.

Whole body magnetic resonance imaging (MRI) was performed 11 days after the operation; it confirmed the recanalisation of the femoral and iliac veins bilaterally, with possible recoil in the distal IVC. It also showed bone and muscle necrosis in the left thigh and leg.

Eighteen days after the intervention, she left hospital with a therapeutic dose of enoxaparin, which was switched to rivaroxaban after three months. After physiotherapy, she recovered fully and was able to go back to normal life without any sequelae. Corticosteroids were progressively decreased after five months and after ruling out the presence of antiphospholipid antibodies anticoagulation was reduced to prophylactic dose for other six months, since the venous flow appeared of excellent quality.

The most recent MRI showed mild persistence of osteonecrosis signs at proximal calf level, with relative sparing of growth plates, and a completely asymptomatic re-occlusion of the infrarenal IVC.

## DISCUSSION

PCD is defined as a fulminant form of proximal venous thrombosis with abolition of the venous outflow, causing severely increased venous pressure, oedema, and compartment syndrome, leading to high arterial resistance and tissue ischaemia. If untreated, this condition, if sufficiently severe, may lead to venous gangrene, with an amputation rate varying 12–68% and a mortality rate of 20–40%.^3^

In the paediatric population, the most important risk factor for the development of VTE is central venous line insertion, and, as in adults, cancer and sepsis also play a significant role.^2^ This review found two case reports of PCD in children aged five years and seven years; both survived.^4^^,^^5^ One underwent catheter directed thrombolysis and had a catastrophic clinical outcome with major limb amputation after several months of hospitalisation. The other underwent pharmacomechanical thrombolysis that eventually required stent implantation because of early re-thrombosis, with favourable six month follow up.

In this case, several concomitant causes came together to cause a massive DVT.

Haemolytic anaemia and high dose corticosteroid therapy, together with atresia of the inferior vena cava, precipitated a condition of stasis and hypercoagulable state.^6^^,^^7^

There are multiple strategies to restore venous outflow, including open surgery, CDT, mechanical thrombectomy (MT), and simultaneous use of thrombolytic agents with MT (pharmacomechanical CDT) or assisted by high intensity ultrasound (EKOS, EkoSonic Endovascular System, Bothell, WA, USA).^8^, ^9^, ^10^, ^11^, ^12^ In this case, MT was preferred over lysis, considering the severity and rapid clinical deterioration. Among the available thrombectomy systems, the Penumbra Lightning aspiration tubing provides intermittent or continuous aspiration when it detects the aspiration catheter is in a patent vascular segment with flow or engaged with thrombus, respectively, thus allowing minimal blood loss, which was essential for this young patient. After re-opening the iliofemoral veins, longstanding stagnation of contrast dye was noted, which motivated the attempt to re-open the IVC. Recanalisation was possible with soft guidewires and support catheters, without sharp manoeuvres, thus supporting the hypothesis of atresia rather than agenesis. Catheter directed thrombolysis minimises systemic exposure and has a high success rate but still exposes the patient to a small (typically <1%) risk of severe bleeding.

Stenting at this age is not advocated because of long term consequences, and even if the treated vena cava is re-occluded, its recanalisation enabled the life threatening situation to be reversed and return to the pre-existing situation. After nine months, she remains asymptomatic, but long term follow up is necessary to ensure that no post-thrombotic syndrome develops. Further recanalisation and stenting of the IVC when she is an adult remain possible if required.

## Conclusion

This review reports an extremely severe case of bilateral phlegmasia with a concomitant IVC atresia in a two year old girl. The patient was successfully treated with a combination of therapies that are seldom documented in children, confirming that modern endovascular techniques for the venous system also apply to the paediatric population. It is hoped that this may help other physicians facing similar life threatening situations.

## FUNDING

None.

## CONFLICT OF INTEREST

None.

## References

[1] Suwanabol P.A., Tefera G., Schwarze M.L. (2010). Syndromes associated with the deep veins: phlegmasia cerulea dolens, May-Thurner syndrome, and nutcracker syndrome. Perspect Vasc Surg Endovasc Ther.

[2] Spentzouris G., Scriven R.J., Lee T.K., Labropoulos N. (2012). Pediatric venous thromboembolism in relation to adults. J Vasc Surg.

[3] Bhatt S., Wehbe C., Dogra V.S. (2007). Phlegmasia cerulea dolens. J Clin Ultrasound.

[4] Kuo I., Smith J., Abou-Zamzam A.M. (2011). A multimodal therapeutic approach to phlegmasia cerulea dolens in a pediatric patient. J Vasc Surg.

[5] Tran J., Rafique Z. (2015). Phlegmasia cerulea dolens in the pediatric population: a life-threatening condition. J Emerg Med.

[6] Shiroshita K., Okayama M., Soma H., Sato Y., Hayashi H., Shiozawa Y. (2023). Thromboembolism early after glucocorticoid administration in patients with autoimmune hemolytic anemia. Clin Hematol Int.

[7] Tufano A., Cannavacciuolo F., Gianno A., Cerbone A.M., Mangiacapra S., Coppola A. (2017). Inferior vena cava agenesis and deep vein thrombosis in the young: a review of the literature and local experience. Semin Thromb Hemost.

[8] Kohi M.P., Kohlbrenner R., Kolli K.P., Lehrman E., Taylor A.G., Fidelman N. (2016). Catheter directed interventions for acute deep vein thrombosis. Cardiovasc Diagn Ther.

[9] Kim H., Labropoulos N., Blake A.M., Desai K. (2022). Prevalence of inferior vena cava anomalies and their significance and impact in clinical practice. Eur J Vasc Endovasc Surg.

[10] Mentesidou L., Dettoraki A., Michalopoulou A., Pergantou H., Malama A., Gavra M. (2023 1). Inferior vena cava agenesis presenting as deep vein thrombosis in an eight year-old girl. Blood Coagul Fibrinolysis.

[11] Reslan O.M., Raffetto J.D., Addis M., Sundick S. (2015). Congenital absence of inferior vena cava in a young patient with iliofemoral deep venous thrombosis treated with ultrasound-accelerated catheter-directed thrombolysis: case report and review of the literature. Ann Vasc Surg.

[12] Tarazi M., Bashir A., Khan K., Kakani N., Murray D., Serracino-Inglott F. (2020). A literature review and case series of DVT patients with absent IVC treated with thrombolysis. Ann Vasc Surg.

